# Exploring the Students’ Perceived Effectiveness of Online Education during the COVID-19 Pandemic: Empirical Analysis Using Structural Equation Modeling (SEM)

**DOI:** 10.3390/bs13070578

**Published:** 2023-07-12

**Authors:** Qamar Ali, Azhar Abbas, Ali Raza, Muhammad Tariq Iqbal Khan, Hasan Zulfiqar, Muhammad Amjed Iqbal, Roshan K. Nayak, Bader Alhafi Alotaibi

**Affiliations:** 1 Department of Economics, Virtual University of Pakistan, Faisalabad 38000, Pakistan; 2 Institute of Agricultural and Resource Economics, University of Agriculture, Faisalabad 38040, Pakistan; 3 College of Life Sciences, Northeast Forest University, Harbin 150080, China; 4 Department of Economics, Government Graduate College, Jaranwala 37200, Pakistan; 5 Division of Agricultural and Natural Resources, University of California, 2801 2nd Street, Davis, CA 95616, USA; 6 Department of Agricultural Extension and Rural Society, College of Food and Agriculture Sciences, King Saud University, Riyadh 11451, Saudi Arabia

**Keywords:** COVID-19 pandemic, internet, learning management system, learning satisfaction, online education, efficiency

## Abstract

The world faced COVID-19, which was a threat to public health and disturbed the educational system and economic stability. Educational institutes were closed for a longer period, and students faced difficulty to complete their syllabus. The government adopted a policy of “suspending classes without stopping learning” to continue education activities. However, student satisfaction with online education is a growing concern. Satisfaction of students is an important indicator of academic quality. Therefore, this study attempts to investigate the influencing factors behind learning satisfaction using information from 335 students from various institutes in Pakistan. This research examined the impact of computer and internet knowledge, instructor and course material, and Learning Management Systems (LMS) on learning satisfaction. The path coefficients were obtained via Partial Least Square-Structural Equation Modeling (PLS-SEM). The LMS is a tool that facilitates the learning process with the provision of all types of educational material. The path coefficient was more in the case of LMS (0.489), which indicates its positive and significant role to attain learning satisfaction. The instructor and course material ordered second (0.261), which shows that the quality of an instructor and course material also plays a positive role to attain learning satisfaction. The computer and internet are essential ingredients of online education, showing a significant and positive path coefficient (0.123), implying that computer and internet knowledge could enhance learning satisfaction. The universities should develop their LMS to implement online education with quality course materials. It is also vital that the instructor should be up to date with modern learning techniques while ensuring internet connectivity, especially in rural areas. The government should provide an internet connection to students at discounted rates.

## 1. Introduction

The outbreak and spread of coronaviruses during 2019–2020 have significantly affected social and economic development [1,2,3]. It is possible to reduce the COVID-19 rate via social distancing and lockdowns. Therefore, educational institutes were closed for a longer period, and students faced difficulty to complete their syllabus. This inconvenience leads to digital intervention in education. Many universities started online education using Microsoft Teams, Blackboard, Zoom, and related platforms [4]. Information and communication technologies (ICT) became a powerful tool that transforms the education sector across the globe. COVID-19 is also responsible for the educational transformation from physical to online [5]. 

Online education has become a well-known learning model among students to continue their education in developed and developing countries. For example, universities in the United States (i.e., Harvard University, Ohio State University, Stanford University, and Princeton University) canceled face-to-face teaching classes and moved to online education [6]. In China, universities shut campuses, moved to online teaching, and offered more than 5000 courses within two weeks. For instance, Zhejiang University (ZJU) introduced an online teaching hub “Learning at ZJU” which involved 570,000 visits, and the live streaming app “DingTalk ZJU” involved 300,000 learners [7]. Turkey also adopted the online teaching method [8,9].

The popularity of online learning and training has been increasing worldwide. It reduced the spatial and temporal problems of conventional education. Online learning is advantageous for students due to learning opportunities and online material at their houses [10]. There has been a rapid increase in online platforms and online courses/resources during the past three years. Currently, distance education has become a rapidly growing form of education [11]. The expected increase in the value of the Learning Management System (LMS) will be 18.44 billion USD by 2025 compared to 5.05 billion USD in 2016, which showed a 15.52% growth in the value of the global LMS. It can increase access to education and training, improve learning quality, and reduce costs [10]. 

In Pakistan, it is difficult for most institutes to manage and start online education. Many government institutes were not able to go online due to scarcity of resources. However, different institutes tried their best to provide continued education to their students [12]. In Pakistan, the role of distance education is vital for the provision of access to obtain higher education. The Virtual University of Pakistan, a federal government university established in 2002, aims to give educational opportunities to those unable to continue traditional education. The Virtual University of Pakistan uses modern ICT to provide quality education [13].

Due to COVID-19, the Higher Education Commission (HEC) directed the universities to use the LMS for online classes. The HEC established the National Knowledge Bank to provide online access to educational materials such as syllabuses, curriculums, links to digital libraries, lists of textbooks, lesson plans, exam questions, video lectures, assignments, and quizzes through LMS [14]. The HEC guided the universities to consult with the Virtual University of Pakistan to obtain technical support for the LMS, including administration, tracking, documentation, reporting, and transmission of educational courses, development or learning programs, and training programs. A student can access the digital content by using the login and password. After signing in, a student can access course content, recorded video lectures, notice boards, assignments, educational content, grades, and fee details [15].

Students can use a computer, laptop, smartphone, iPad, and other electronic devices for online education. The use of information technology helps deliver instruction and content to the students. Online education needs the instructor’s time for the development of the course. After the development of a course, the instructor can reply to students’ queries by email and involvement in discussion boards, mark assignments, and regularly update the online course. There are different formats of online education, such as online learning management systems (LMS). Online education includes blogs, chatrooms, discussion boards, podcasts, wikis, video lecture, learning objects, web conferencing, and wireless mobile devices. However, it is challenging for educators to keep in touch with advanced technologies and use new teaching techniques to attain more student engagement [16]. The use of new educational technologies is beneficial for students and professors. In online teaching, a course is conducted using the internet, and online courses become valuable for students who cannot enroll in full-time conventional education. Online education showed different benefits for society [17].

Online education is considered an alternative teaching method, especially during the COVID-19 pandemic. However, student satisfaction with online education is a growing concern. Satisfaction of students is an important indicator of academic quality [18]. Student satisfaction is a subjective perception of students about the success of a learning environment [19].

Yet, studies that examine the impact of online teaching on students are limited and lack consensus. Thus, it is also required to assess online education students’ learning satisfaction. Therefore, this study makes three main contributions. First, it assesses the role of computer and internet knowledge in the learning process. Second, this study highlights the significance of tutors and reading material in the learning process. Third, it extends the literature by considering the development of an LMS and its benefits in the learning process, especially during the ongoing COVID-19 pandemic. 

## 2. Brief Literature Review

Online education is a cost-effective, flexible, and self-pacing way of learning that increases the access of students to resources [20]. Different studies examined the factors that influenced students’ satisfaction with e-learning [21]. Patrinos and Shmis [9] showed the impact of COVID-19 on the education system of Central Asia and Europe. Many countries introduced precautionary measures in education systems such as the temporary closing of educational institutions. However, China ensures the continuity of the educational process by introducing electronic textbooks and online courses. Many countries in Central Asia and Europe also directed their education department to close their educational institutions. Online education in Central Asia and Europe depends upon the availability of online content, devices, and internet connectivity at home. The students who have financial resources can afford computers and multiple devices, but poor students can hardly afford such devices and internet connections. If the situation closer to educational institutes lasts for months, then it is required to start online education by (a) ensuring the equipment and internet connectivity for vulnerable students, (b) improving the connectivity of schools, and (c) improving the financing of digital educational content. 

According to Khaliq [8], South Korea will switch to online education for its students who have been at home because of COVID-19. In South Korea, the Ministry of Education declared the start of online lessons for students up to high school by 9 April 2020. The new academic session, scheduled to start in March 2020, was delayed due to the pandemic for five weeks. Turkey also adopted the online teaching method due to this outbreak. Abdalla [6] reported that universities in the United States canceled classes and switched to online education due to COVID-19. The closure of educational institutions was observed in those states which are hardest hit by COVID-19 such as New York, California, and Washington. Harvard University in Cambridge, Massachusetts, directed their students to stay at home, and they are now engaged in virtual teaching. Princeton University, New Jersey, announced that the courses, seminars, and lectures would be moved online. Stanford University, California, and Ohio State University also stopped face-to-face teaching.

Kornpitack and Sawmong [18] assessed the factors influencing student satisfaction in Thailand. Different significant factors were performance expectancy, learner interaction, actual use, and behavioral intention. Satyawan et al. [22] reported the success of Undiksha E-Learning COVID-19 in Indonesia. The concentration of students was 96% (paying attention to lectures) with the use of Undiksha E-Learning. Similarly, the understanding of learning material was 88%, and the motivation of students in online learning was 77%. Iqbal et al. [23] studied the experiences and perceptions of students considering different aspects of online learning in Pakistan. The participants showed concerns about the quality of online instructions and the lack of institutional support. Moreover, barriers to online education were electricity crises, inappropriate study environments, and internet issues. However, most of the students were satisfied with online classes. Mohammed et al. [21] explored the factors behind students’ satisfaction with online education during the pandemic in Malaysia. Results showed four significant factors behind students’ satisfaction such as (a) system quality, (b) instructor’s performance, (c) student factors, and (d) course evaluation. 

## 3. Methodology

### 3.1. Study Area and Data

This study is based on primary data collection, which was collected using different data collection instruments. These instruments are different tools which are used to collect these data. The data collection instruments are online questionnaires, observations, questions, and statistical tests. The online questionnaire was used to assess the influential factors behind the learning satisfaction of students during the COVID-19 pandemic. Therefore, 335 students from different academic programs in Pakistan completed online questionnaires from 15 April 2021 to 30 June 2021. The questionnaire has been validated by the academic staff. The measurement items (i.e., questions) of each latent variable were validated by different statistical tests. Similarly, different validity of latent variables was also check by different statistical tests. Learning satisfaction (endogenous variables) is the objective of online education, especially during COVID-19. The students answered each question on a five-point Likert scale (5 = strongly agree, 4 = agree, 3 = uncertain, 2 = disagree, and 1 = strongly disagree). According to Rasoolimanesh et al. [24], a sample size of 100 is satisfactory to apply the Partial Least Square Structural Equation Model (PLS-SEM). Therefore, the sample size of the current study was appropriate to investigate the drivers of learning satisfaction empirically.

### 3.2. Measuring Constructs/Instruments and Hypothesis

Due to the COVID-19 pandemic, governments of different countries directed educational institutions to start online education. It is an alternate teaching technique under such conditions. However, it is also essential to consider students’ feedback about this learning method. Therefore, it is required to assess the learning satisfaction of students. It is also required to examine the influencing factors behind learning satisfaction. Therefore, this research examines the effect of computer and internet knowledge (CIK), instructor and course material (ICM), and Learning Management System (LMS) on learning satisfaction (LS), using the following hypothesis:

**H1:**  *Computer and internet knowledge have a positive impact on learning satisfaction*.

**H2:**  *Instructor and course materials have a positive impact on learning satisfaction*.

**H3:**  *The LMS has a positive impact on learning satisfaction*.

The construct is a complex variable that involves several related factors. For appropriate results, it is recommended to assess the validity of each construct using convergent validity [25], such as factor loading, Average Variance Extracted (AVE), and Composite Reliability (CR) [26]. The factor loading value for each indicator was estimated using Confirmatory Factor Analysis. It required that the loading value should be more than 0.7. However, the factor loading above 0.5 is also good [27]. The reliability of measuring constructs requires that Cronbach’s alpha ≥ 0.7 [25]. Moreover, a construct is reliable if CR ≥ 0.7 [27]; thus, its value is estimated as [25]:

(1) $$CR=\frac{{\left(\sum_{i=1}^{n}\lambda_{yi}\right)}^{2}}{{\left(\sum_{i=1}^{n}\lambda_{yi}\right)}^{2}+\left(\sum_{i=1}^{\rho }var\left(\varepsilon_{i}\right)\right)}$$

where var(ε_i_) depicts the variance and λ_yi_ depicts standardized factor loading [28].

Fourthly, the reliability of the construct was tested by AVE, which reflects the variance captured by a construct and variance due to measurement error. The AVE value is reliable if AVE ≥ 0.7. However, the AVE ≥ 0.5 is satisfactory [27]. The AVE is estimated as [25]:

(2) $$AVE=\frac{\sum_{i=1}^{n}{\lambda_{i}}^{2}}{n}$$

where λ_i_ is standardized factor loading, and n indicates the number of items [28].

It is needed to check the significant variance among several constructs using the discriminant validity method, which indicates the difference between different constructs. For two constructs, discriminating validity is examined by comparing their AVE scores with their squared correlation. For each construct, the square root of AVE should be more than the correlations of that specific construct with the others [28].

### 3.3. The Partial Least Square-Structural Equation Model (PLS-SEM)

The PLS-SEM is widely used for regression analysis [29], and it is appropriate to investigate the complex relationships between latent variables [30,31]. The PLS-SEM is a multivariate model for the simultaneous estimation of all structural paths between the latent variables [32]. The endogenous variables are unobserved variables measured by using different observed variables [29,33]. Latent constructs are unobserved concepts such as learning satisfaction, estimated using various indicators (survey questions). It is a two-stage method, involving (1) estimation of latent variables score and outer loadings to measure constructs through several iterative steps [27] and (2) path coefficients estimation among latent variables via ordinary least squares (OLS) [34]. Its empirical equation is [28,35]:

(3) $$Y=\beta_{0}+\beta_{1}X_{1}+\beta_{2}X_{2}+\varepsilon_{1}$$

The PLS-SEM does not require strong assumptions about sample size, distribution, and measurement scale [27]. The estimation is based on a non-parametric bootstrapping technique [32], which reveals probability scores to confirm path coefficient stability [36]. The proposed hypotheses were tested with the help of path coefficients [31]. The reliability of the model was also confirmed by the goodness of fit (GoF) value, estimated using the geometric mean of AVE and R^2^ for the endogenous construct [29,37]:

(4) $$GoF=\sqrt{\bar{AVE}\times \bar{R^{2}}}$$

The baseline cut-off GoF values are 0.1 (small sample), 0.25 (medium sample), and 0.36 (large sample) [37].

## 4. Results

### 4.1. Characteristics of Respondents

Table 1 reveals the demographic characteristics of the student. A maximum of 60.30% of students belonged to the 20–30 age group, while 55.52% of respondents were male. About 58.81% of respondents have enrolled students of BS (16 years), followed by M.Phil (20.30%), Ph.D. (15.82%), Intermediate (2.69%), and Matriculations (2.39%). The Internet connectivity was very good for 27.46% of students and good for 59.40% of students. More than 13% of students faced difficulty with internet connectivity. Mostly 34.93% of students liked online education, followed by a mix of conventional and online classes (24.48%), entirely conventional classes (20.60%), and more online with some on-campus activities (20%). 

### 4.2. Response of Participants

To assess the usefulness of online education during COVID-19, Table 2 and Table 3 show the frequency-wise responses for each question. Table 2 shows that 83.58% of participants know about online education. About one-third of participants (73.73%) reported that they know about computers. Similarly, 77.31% of participants know about the use of Internet facilities. About 89.55% of students confirmed they want online classes during COVID-19. About 88.96% of students feel that online learning will help them in their studies. Similarly, 85.67% of students considered that online learning is a good alternative to conventional learning. The LMS is effective in online education, but only 54.93% of students know about the LMS. It shows the use of LMS is less in Pakistan. Similarly, only 42.09% of students know about Virtual University Labs/Mobile Labs. 

Table 3 shows the response to questions used in constructing latent variables in the estimation of path coefficients. More than 80% of respondents agreed and strongly agreed that they are familiar with the basic functions of Microsoft Office (Word, Excel, and PowerPoint). However, about 72% of respondents (agreed and strongly agreed) confirmed that they are confident in using online learning software, i.e., Skype, ZOOM, and Google Meet. Approximately, 80% of participants confirmed that their teachers were actively involved in facilitating good education. About 77% of participants strongly agreed and agreed that their teacher provided timely and helpful feedback on assignments, exams, and queries. However, about 70% of respondents (strongly agreed and agreed) claimed that the course material was organized into logical and understandable components. About 70% of participants strongly agreed and agreed with the statement that the overall use of online education/LMS is good. However, only 60% of respondents strongly agreed and agreed with the statement that online education quality is equivalent to face-to-face education.

### 4.3. Assessment of Validity of the Measurement Model

The model used different latent variables such as computer and internet knowledge, instructor and course material, LMS, and learning satisfaction. Before estimating path coefficients, it is required to validate these latent variables. Several tests reflect the reliability of selected instruments (questions) and latent constructs. Results (Table 4) indicate that the loading value is more than 0.7 for each question, which indicates the reliability of all constructs. The reliability was also tested through CR value, which is more than 0.7 for each latent. Moreover, the reliability was also tested using the AVE score, which should be greater than 0.5. The AVE score for each construct was higher than 0.5, showing the reliability of these constructs. The internal consistency was also tested using Cronbach-α, and ρ-A, which shows an acceptable range (0.7–0.95) [38]. After validating latent variables, the path coefficients using PLS-SEM is the next step.

Before path coefficient estimation, it is also required to check the difference between selected constructs. Therefore, the difference between the two latent variables was tested using discriminative validity [38]. The square root of the AVE for each latent variable (Table 5) in a bold diagonal is higher than the correlation score for all other latent variables. For example, the AVE value for LS was 0.710, whose square root was 0.843 (bold diagonal). The square root of the AVE for LS was higher than the correlation score with all other latent variables. Therefore, discriminant validity analysis confirmed a significant difference between selected constructs. 

### 4.4. Partial Least Square (PLS) Regression

**Figure 1:**
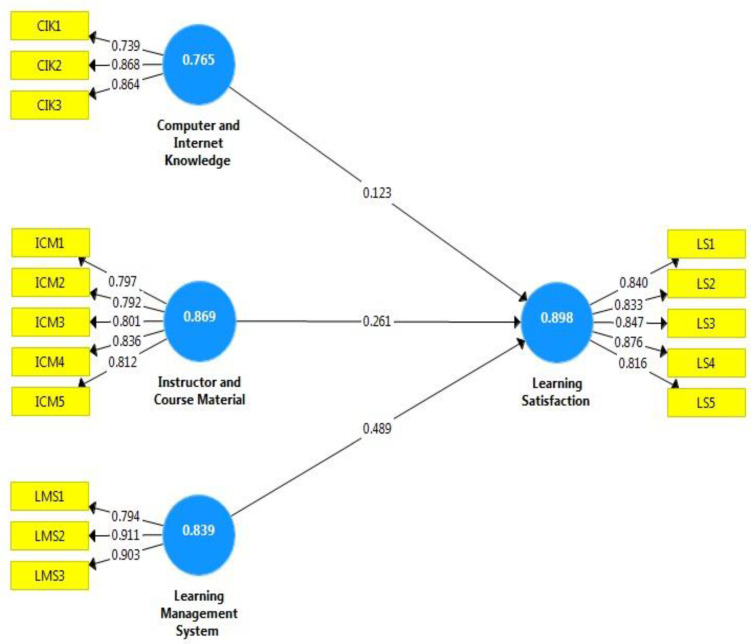
Results of the PLS-SEM.

The pandemic was responsible for the closure of educational institutes for a long period. It is required to start online education by (a) ensuring the equipment and internet connectivity for vulnerable students, (b) improving the connectivity of schools, and (c) improving the financing of digital educational content. Therefore, this study empirically investigated the impact of computer and internet knowledge, instructor and learning material, and LMS on learning satisfaction. Table 6 shows the path coefficients using the PLS-SEM method. Results confirmed all hypotheses and demonstrated that all the influencing factors significantly impact the learning satisfaction of students during COVID-19. The endogenous variable was learning satisfaction. Computer and internet knowledge, instructor and course material, and learning management system were treated as exogenous variables. Learning Management System (LMS) shows a higher path coefficient (0.489), which shows the role of LMS in learning satisfaction. The instructor and course material ordered a second (0.261), which shows the quality of the instructor and course material also acts as a driver to attain learning satisfaction. The computer and internet are essential ingredients of online education, showing a significant path coefficient (0.123), which shows that computer and internet knowledge could be beneficial for students learning satisfaction. To investigate the reliability of the model, the R^2^ value depicts variance for each endogenous construct and validates the prediction power of the model. Better estimation requires that R^2^ ≥ 0.25 [39,40]. In the current study, the R^2^ was 0.617; thus, R^2^ > 0.25, indicating the prediction capability of the PLS-SEM. The reliability of the model was also tested by the goodness of fit (GoF), which is 0.662. The GoF score was higher than the cut-off value for a large sample (0.36). Figure 1 shows the PLS-SEM framework, indicating different factors that could enhance the learning satisfaction of students. It contains measurement indicators (questions) for latent variables in the yellow boxes. Loading values for each indicator are evident between yellow boxes and blue circles. The value inside a blue circle is Cronbach-α, showing each construct’s reliability. The path coefficients are values between two blue circles, which are all significant. 

## 5. Discussion

Traditional teaching methods engage intelligent students as well as students having difficulty learning. However, e-learning is an effective tool, which is helpful for teachers due to several benefits (a) accessibility, (b) time-saving, (c) cost-effectiveness, (d) better connection, (e) better parent–teacher communication, (f) efficiency, (g) flexibility, (h) innovation, and (i) passion-based learning [41]. Universities with better infrastructure are successful at implementing E-Learning. The literature shows that there are many influential factors behind the success of learning management systems such as access to electricity, internet connection, economic status, and ICT skill according to Mohammadi et al. [42]. The impact of LMS was positive on learning satisfaction, which implies that universities should successfully develop their LMS to implement online education, especially during emergencies. In Albania, there was a positive connection between LMS self-efficacy and students’ satisfaction [19]. Similarly, the impact of the instructor and course material is positive which is the driver of learning satisfaction. The satisfaction of students is higher in the presence of high-quality connections with instructors. Timely feedback from teachers is important in the absence of face-to-face learning [18]. The knowledge of computers and the internet is important to increase learning satisfaction. Iqbal et al. [23] also mentioned that the satisfaction level was higher for learners who had good knowledge of educational tools and technologies. According to Kornpitack and Sawmong [18], ICT is essential to conduct online education. The level of student satisfaction was positively related to the utilization of effective educational tools, student–teacher communications, course content, and presentation skills of instructors [23]. A well-established internet connection is needed for the success of online learning. Thus, the government should provide an internet connection to students at discounted rates. 

## 6. Conclusions and Policy Implications

The increase in the spread of coronaviruses was observed worldwide, which significantly affects social and economic development. Educational institutes were closed for a longer period, and students faced difficulty to complete their syllabus. The government adopted a policy of “suspending classes without stopping learning” to continue education activities. However, student satisfaction with online education is a growing concern. Satisfaction of students is an important indicator of academic quality. Therefore, this study investigated the influencing factors behind the learning satisfaction of students. The LMS had a greater path coefficient (0.489), which indicates the role of LMS in the increase in learning satisfaction. Development of LMS at the institutional level is recommended to continue the learning process in off-hours/lock-down, especially during COVID-19. The instructor and course material ranked second (0.261), which implies that the quality of an instructor and course material also acts as a driver to increase learning satisfaction. Therefore, it is required to enhance the quality of the course material continuously. It is also vital that the instructor should be up to date with modern learning technologies. Computers and the internet have a key role in the learning process, especially in the recent era. Thus, the government should ensure internet connectivity, especially in rural areas. The student in the rural area had traveling difficulties and could not meet the living expenses in a city. Therefore, the government should provide subsidies for an internet connection to students, especially in rural areas. Moreover, as the findings of this work are intuitive with greater policy insights, regarding the infrastructure especially the learning–teaching one to be phased out to cater needs of the students located remotely. This option can have multiple benefits: attracting students to advanced stages of education, creating interest and a sense of responsibility both for teaching and learning through maintenance of track and progress with the support of online teaching gadgets and software, and improving efficiency both on the part of students and teachers through improved communication, recall, and feedback mechanisms. Nevertheless, the bottlenecks such as power and connection outages need to be given due consideration through the availability of alternative energy provision. In the case of Pakistan, the system of online education in the aftermath of the peak COVID-19 pandemic has effectively taken its course and is now being featured in different ways of linkages among academia, industry, and stakeholders.

This research has some limitations due to a shortage of time and space. First, it assesses the effectiveness of online education by considering students across Pakistan. There is heterogeneity in the online education system across several universities or institutions. Thus, future studies should perform a comparison of online education by selecting different educational institutes in Pakistan. Second, this study assesses the impact of only three selected constructs on learning satisfaction. Therefore, future studies should add more latent constructs to the path-coefficients estimation. 

## Figures and Tables

**Table 1:** Profile of respondents (N = 335).

Demographic Status	Frequency (n)	%
Age (years)		
Below 20	47.00	14.03
20–30	202.00	60.30
31–40	73.00	21.79
Above 40	3.88	3.88
Gender		
Male	186.00	55.52
Female	149.00	44.48
Class		
PhD	53.00	15.82
M.Phil (18 years)	68.00	20.30
M.A./BS (Hons). (16 years)	197.00	58.81
Intermediate	9.00	2.69
Matriculations	8.00	2.39
Internet connectivity in your area		
Very Good	92.00	27.46
Good	199.00	59.40
Not Good	42.00	12.54
Not Available	2.00	0.60
Family Income (Rs./Month)		
Less than 25,000	83.00	24.78
25,001–50,000	102.00	30.45
50,001–75,000	47.00	14.03
76,001–100,000	62.00	18.51
Above 100,000	41.00	12.24
Which type of course delivery mode do you prefer?		
Blended (More Online with some on-campus activities)	67.00	20.00
Entirely conventional (classroom only)	69.00	20.60
Mix of conventional and online classes	82.00	24.48
Online	117.00	34.93

**Table 2:** The response of participants (percentage).

Questions	Yes	A Little Bit	No
Do you know about online education?	83.58	11.04	5.37
Do you know how to operate a computer?	73.73	22.99	3.28
Do you know how to use the internet facility?	77.31	21.19	1.49
Can you afford internet expenditures?	90.15	0.00	9.85
Do you want online classes during the COVID-19 epidemic lockdown to enhance your learning?	89.55	0.00	10.45
Is your university more concerned about your current semester/study during the COVID-19 pandemic?	94.33	0.00	5.67
Are you getting regular assignments while sitting at home?	76.72	0.00	23.28
Do you feel you are fully prepared for online learning?	83.88	0.00	16.12
Do you think online learning will help you in your studies?	88.96	0.00	11.04
Do you think online learning is a good alternative to conventional learning?	85.67	0.00	14.33
Do you know about the Virtual University of Pakistan provides Online Education?	64.78	25.37	9.85
Do you know about the LMS (Learning Management System)?	54.93	28.36	16.72
Do you know about Virtual University Labs/Mobile Labs?	42.09	24.78	33.13

**Table 3:** Response (Likert Scale) of participants (%).

Constructs/Measurement Items	Strongly Disagree	Disagree	Uncertain	Agree	Strongly Agree
Computer and Internet Knowledge (CIK)					
CIK1: I am familiar with the basic functions of Microsoft Office (Word, Excel, and PowerPoint)	0.90	2.99	11.34	47.46	37.31
CIK2: I feel confident in using online learning software, i.e., Skype, ZOOM, and Google Meet	2.09	6.87	17.91	43.28	29.85
CIK3: I feel confident in using the internet to find or gather information for online learning.	1.49	3.88	8.96	50.45	35.22
Instructor and Course Material (ICM)					
ICM1: The teachers are actively involved in facilitating good Education	2.09	3.58	15.52	49.55	29.25
ICM2: The teachers are responsive to students’ concerns.	0.60	4.48	16.42	48.36	30.15
ICM3: The teacher provides timely and helpful feedback on assignments, exams, and queries.	1.49	6.27	14.93	52.24	25.07
ICM4: The course objectives and study materials are communicated.	2.39	8.96	17.91	47.16	23.58
ICM5: The course material was organized into logical and understandable components	2.39	9.55	17.31	49.25	21.49
Learning Management System (LMS)					
LMS1: The overall usability of the online education/LMS is good.	1.49	9.25	17.61	40.90	30.75
LMS2: Online education quality is equivalent to face-to-face courses I have taken.	10.15	13.73	15.82	37.01	23.28
LMS3: The quality of learning in online courses is better than in face-to-face courses.	12.84	16.12	17.31	32.84	20.90
Learning Satisfaction (LS)					
LS1: Online learning enables me to obtain more learning resources.	5.37	10.15	18.81	44.78	20.90
LS2: Online learning provides sufficient discussion opportunities.	6.57	11.04	23.58	38.21	20.60
LS3: Online learning enables me to learn at any time and location of my choice.	3.88	5.97	13.73	44.18	32.24
LS4: Online learning enables me to review learning materials repeatedly.	2.99	8.66	16.42	45.37	26.57
LS5: Online learning can help to broaden my general knowledge.	4.48	9.25	18.81	42.39	25.07

**Table 4:** Model assessment.

Constructs/Measurement Items	Loading	Cronbach-α	ρ-A	CR	AVE
Computer and Internet Knowledge (CIK)					
CIK1	0.739	0.765	0.779	0.865	0.682
CIK2	0.868				
CIK3	0.864				
Instructor and Course Material (ICM)					
ICM1	0.797	0.869	0.884	0.904	0.652
ICM2	0.792				
ICM3	0.801				
ICM4	0.836				
ICM5	0.812				
Learning Management System (LMS)					
LMS1	0.794	0.839	0.854	0.904	0.758
LMS2	0.911				
LMS3	0.903				
Learning Satisfaction (LS)					
LS1	0.840	0.898	0.899	0.924	0.710
LS2	0.833				
LS3	0.847				
LS4	0.876				
LS5	0.816				

**Table 5:** Discriminant validity and correlations.

Constructs	LS	LMS	ICM	CIK
LS	**0.843**			
LMS	0.749	**0.871**		
ICM	0.684	0.707	**0.808**	
CIK	0.589	0.616	0.634	**0.826**

**Table 6:** PLS-SEM estimation.

Hypothesis	Hypothesized Path	Path Coefficients	Standard Error	T-Stat.	Prob.	Decision	Driver/Barrier
Total Effects							
H1	CIK→LS	0.123 *	0.059	2.080	0.038	Supported	Driver
H2	ICM→LS	0.261 **	0.064	4.096	0.000	Supported	Driver
H3	LMS→LS	0.489 **	0.057	8.564	0.000	Supported	Driver
The goodness of fit (Model)							
R^2^	0.617	Adj. R^2^	0.613	Goodness of fit (GoF)		0.662 (model is good)	

Note: * and ** show level of significance at 5% and 1%, respectively.

## Data Availability

Data will be made available on request.

## References

[1] Coccia M. (2021). The relation between length of lockdown, numbers of infected people and deaths of COVID-19, and economic growth of countries: Lessons learned to cope with future pandemics similar to COVID-19 and to constrain the deterioration of economic system. Sci. Total Environ..

[2] Inegbedion H. (2021). Impact of COVID-19 on economic growth in Nigeria: Opinions and attitudes. Heliyon.

[3] Kumar V., Alshazly H., Idris S.A., Bourouis S. (2021). Evaluating the Impact of COVID-19 on Society, Environment, Economy, and Education. Sustainability.

[4] Muthuprasad T., Aiswarya S., Aditya K.S., Jha G.K. (2021). Students’ perception and preference for online education in India during COVID-19 pandemic. Soc. Sci. Humanit. Open.

[5] Ulum H. (2022). The efects of online education on academic success: A meta-analysis study. Educ. Inf. Technol..

[6] Aljazeera US Universities Switch to Online Courses Due to Coronavirus. https://www.aljazeera.com/news/2020/3/10/us-universities-switch-to-online-courses-due-to-coronavirus.

[7] World Economic Forum. https://www.weforum.org/agenda/2020/03/coronavirus-china-the-challenges-of-online-learning-for-universities/.

[8] Khaliq R.U.S. Korea Switches to E-Education Amid COVID-19 Pandemic. https://www.aa.com.tr/en/asia-pacific/skorea-switches-to-e-education-amid-covid-19-pandemic/1786750#!.

[9] Patrinos H.A., Shmis T. (2020). Can Technology Help Mitigate the Impact of COVID-19 on Education Systems in Europe and Central Asia?.

[10] Panigrahi R., Srivastava P.R., Sharma D. (2018). Online learning: Adoption, continuance, and learning outcome—A review of literature. Int. J. Inf. Manag..

[11] Yang C., Xu D. (2023). Predicting student and instructor e-readiness and promoting e-learning success in online EFL class during the COVID-19 pandemic: A case from China. PLoS ONE.

[12] Farrukh M., Soomro T.R., Ghazal T.M., Alzoubi H.M., Alshurideh M., Alshurideh M., Al Kurdi B.H., Masa’deh R., Alzoubi H.M., Salloum S. (2023). Perspectives of Online Education in Pakistan: Post-COVID Scenario. The Effect of Information Technology on Business and Marketing Intelligence Systems.

[13] Saghir S., Zahid K., Tabassum A., Iqbal Z., Ahmad M., Liaquat H. (2016). Challenges in the Online Learning of Economics. ASEAN J. Open Distance Learn..

[14] The News. HEC Asks Universities to Start Online Teaching. https://www.thenews.com.pk/print/636738-hec-asks-universities-to-start-online-teaching.

[15] The Dawn. HEC Tells Universities to Go for Online Classes. https://www.dawn.com/news/1544000.

[16] Hammerling J.A. (2012). Best practices in undergraduate clinical laboratory science online education and effective use of educational technology tools. Lab. Med..

[17] Stan L.C. (2014). Online Teaching Technique in Maritime Learning Process. Procedia-Soc. Behav. Sci..

[18] Kornpitack P., Sawmong S. (2022). Empirical analysis of factors influencing student satisfaction with online learning systems during the COVID-19 pandemic in Thailand. Heliyon.

[19] Prifti R. (2022). Self–efficacy and student satisfaction in the context of blended learning courses. Open Learn. J. Open Distance e-Learn..

[20] Edu T., Negricea C., Zaharia R., Zaharia R.M. (2022). Factors influencing student transition to online education in the COVID 19 pandemic lockdown: Evidence from Romania. Econ. Res.-Ekon. Istraživanja.

[21] Mohammed L.A., Aljaberi M.A., Amidi A., Abdulsalam R., Lin C.-Y., Hamat R.A., Abdallah A.M. (2022). Exploring factors affecting graduate students’ satisfaction toward E-Learning in the era of the COVID-19 crisis. Eur. J. Investig. Health Psychol. Educ..

[22] Satyawan I.M., Wahjoedi W., Swadesi I.K.I. (2021). The effectiveness of online learning through Undiksha ELearning during the COVID-19 pandemic. J. Educ. Technol..

[23] Iqbal S.A., Ashiq M., Rehman S.U., Rashid S., Tayyab M. (2022). Students’ perceptions and experiences of online education in Pakistani universities and higher education institutes during COVID-19. Educ. Sci..

[24] Rasoolimanesh S.M., Ali F., Jaafar M. (2018). Modeling residents’ perceptions of tourism development: Linear versus non-linear models. J. Destin. Mark. Manag..

[25] Ghadi I., Alwi N.H., Bakar K.A., Talib O. (2012). Construct validity examination of critical thinking dispositions for undergraduate students in University Putra Malaysia. High. Educ. Stud..

[26] Fornell C., Larcker D.F. (1981). Evaluating structural equation models with unobservable variables and measurement error. J. Mark. Res..

[27] Hair J.F., Black W.C., Babin B.J., Anderson R.E. (2010). Multivariate Data Analysis: Global Edition.

[28] Raza A., Ali Q., Hussain T. (2021). Role of knowledge, behavior, norms, and e-guidelines in controlling the spread of COVID-19: Evidence from Pakistan. Environ. Sci. Pollut. Res..

[29] Assemi B., Jafarzadeh H., Mesbah M., Hickman M. (2018). Participants’ perceptions of smartphone travel surveys. Transp. Res. Part F Traffic Psychol. Behav..

[30] Fernández-Heredia Á., Monzón A., Jara-Díaz S. (2014). Understanding cyclists’ perceptions, keys for a successful bicycle promotion. Transp. Res. Part A Policy Pract..

[31] Chung Y., Kim H. (2015). Deep subterranean railway system: Acceptability assessment of the public discourse in the Seoul Metropolitan Area of South Korea. Transp. Res. Part A Policy Pract..

[32] Hair J.F., Hult G.T.M., Ringle C.M., Sarstedt M. (2017). A Primer on Partial Least Squares Structural Equation Modeling (PLS-SEM).

[33] Wolf A., Seebauer S. (2014). Technology adoption of electric bicycles: A survey among early adopters. Transp. Res. Part A Policy Pract..

[34] Lohmöller J.-B. (2013). Latent Variable Path Modeling with Partial Least Squares.

[35] Kock N. (2010). Using WarpPLS in e-collaboration studies: An overview of five main analysis steps. Int. J. e-Collab. IJeC.

[36] Andreev P., Heart T., Maoz H., Pliskin N. Validating formative partial least squares (PLS) models: Methodological review and empirical illustration. Proceedings of the International Conference on Information Systems, ICIS 2009.

[37] Wetzels M., Odekerken-Schröder G., Van Oppen C. (2009). Using PLS path modeling for assessing hierarchical construct models: Guidelines and empirical illustration. MIS Q..

[38] Elmustapha H., Hoppe T., Bressers H. (2018). Consumer renewable energy technology adoption decision-making; comparing models on perceived attributes and attitudinal constructs in the case of solar water heaters in Lebanon. J. Clean. Prod..

[39] Chin W.W. (2009). How to write up and report PLS analyses. Handbook of Partial Least Squares: Concepts, Methods and Applications.

[40] Davison A.C., Hinkley D.V. (1997). Bootstrap Methods and Their Application.

[41] Cioruța B.-V., Lauran M., Coman M., Pop A.L., Lauran A. (2021). About the Benefits of Adopting E-Learning in the Current Romanian Educational System. Asian J. Educ. Soc. Stud..

[42] Mohammadi M.K., Mohibbi A.A., Hedayati M.H. (2021). Investigating the challenges and factors influencing the use of the learning management system during the COVID-19 pandemic in Afghanistan. Educ. Inf. Technol..

